# Interplay between lipid droplets and alpha-synuclein: implication in Parkinson’s disease pathogenesis

**DOI:** 10.3389/fnmol.2025.1681039

**Published:** 2025-12-01

**Authors:** Walid Idi, Razan Sheta, Abid Oueslati

**Affiliations:** 1 CHU de Québec Research Center, Axe Neurosciences, Quebec City, QC, Canada; 2 Department of Molecular Medicine, Faculty of Medicine, Université Laval, Quebec City, QC, Canada

**Keywords:** lipid droplets, alpha-synuclein, Parkinson’s disease, lipid droplet functions, lipid metabolism, lipid-alpha-synuclein interaction, protein aggregation

## Abstract

Lipid droplets (LDs), once considered inert lipid stores, are now recognized as active regulators of lipid metabolism, stress responses, and protein quality control in the brain. Their dysregulation is increasingly linked to neurodegenerative diseases, notably Parkinson’s disease (PD). This review explores the emerging bidirectional relationship between LDs and α-synuclein (α-Syn), a key pathological hallmark of PD. α-Syn can promote LD accumulation by modulating lipid metabolism and inhibiting lipolysis, while LDs can facilitate α-Syn aggregation through specific lipid-protein and membrane interactions. We summarize current evidence on LD structure, function, and dynamics in neuronal and glial cells, and discuss how alterations in lipid composition, oxidative stress, and associated proteins contribute to PD pathology. Understanding the LD-α-Syn interplay reveals new avenues for therapeutic strategies aimed at restoring lipid homeostasis, enhancing LD turnover, and reducing α-Syn toxicity.

## 1 Introduction

Parkinson’s disease (PD) is a progressive neurodegenerative disorder characterized by the selective loss of dopaminergic neurons in the substantia nigra (Surmeier, 2018). A defining pathological feature of PD is the aggregation of α-synuclein (α-Syn) into Lewy bodies (LBs) (Baba et al., 1998).

The implication of α-Syn in PD was first reported in 1997, when a missense mutation in the *SNCA* gene was identified as the cause of a rare familial form of PD (Polymeropoulos, 1997). In addition, LBs and Lewy neurites in idiopathic PD were found to be immunoreactive for α-Syn (Spillantini et al., 1997).

Although the role of α-Syn in PD pathogenesis has been extensively studied, increasing evidence highlights the importance of lipid metabolism and organelle dysfunction in disease progression (Fanning et al., 2020; Galvagnion, 2017; Shahmoradian et al., 2019). Among these organelles, lipid droplets (LDs) have emerged as key players in neuronal health, exhibiting functions beyond energy storage, including lipid homeostasis, stress response, and interactions with misfolded proteins (Farese and Walther, 2009; Girard et al., 2021; Ralhan et al., 2021). These dynamic cytoplasmic structures, composed of a neutral lipid core (triglycerides and sterol esters) encased in a phospholipid monolayer, have emerged as unexpected yet pivotal players in neuronal health and disease (Olzmann and Carvalho, 2019; Walther and Farese, 2012; Zhang L. et al., 2025).

Under physiological conditions, LDs in the brain are primarily found in glial cells, where they mitigate lipid toxicity and help sustain neuronal function (Ralhan et al., 2021; Zhang L. et al., 2025). However, recent studies confirm their presence in neurons during metabolic stress, aging, or neurodegenerative states (Farmer et al., 2020; Goodman et al., 2024; Marschallinger et al., 2020).

Growing research supports a compelling mechanistic link between LDs and α-Syn, revealing a bidirectional relationship. On one hand, α-Syn has been shown to drive the formation of LDs, potentially by modulating lipid metabolism and cellular stress pathways (Girard et al., 2021; Makasewicz et al., 2021). On the other hand, LDs have been shown to promote the pathological aggregation of α-Syn, suggesting that disruptions in lipid homeostasis may actively contribute to the progression of PD (Brekk et al., 2020; Eubanks et al., 2025; Russo and Riessland, 2024).

This review explores the LD-α-Syn axis as a critical driver of PD pathogenesis. Reporting on evidence that LD dynamics, governed by lipid composition, perilipin proteins, and stress-responsive pathways, directly modulate α-Syn toxicity. It also delves into the interplay between LDs and α-Syn, shedding light on how these interactions may contribute to the pathogenesis of PD. The review will also discuss the potential mechanisms linking LD dynamics with α-Syn aggregation, their implications on neuronal health, and how targeting this relationship could open new therapeutic avenues for PD.

## 2 Lipid droplet structure, composition and functionality

### 2.1 Structure and composition

**Figure 1:**
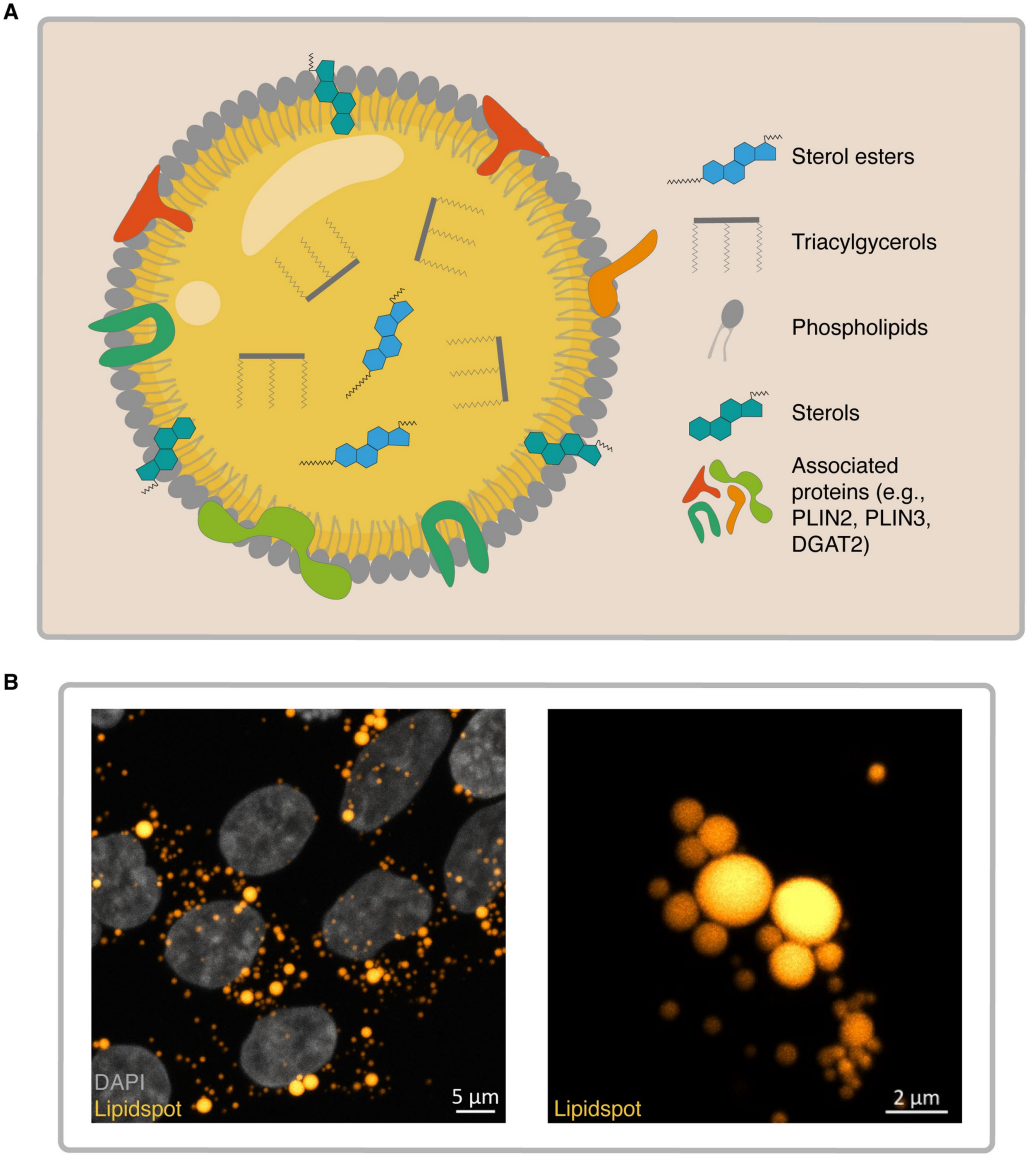
Lipid droplet (LD) composition and structure. **(A)** Schematic representation of a LD. The core consists of neutral lipids, including triacylglycerols and sterol esters, while the monolayer membrane is composed of phospholipids, sterols, and associated proteins. **(B)** Confocal images of LDs in HEK293T cells stained with LipidSpot™ (gold-yellow) after treatment with oleic acid (OA; a monounsaturated fatty acid commonly used to induce lipid droplet formation) (100 μM-24 h). The left panel displays cells counterstained with DAPI to label the nucleus. Scale bar left: 5 μm and right: 2 μm.

LDs are ubiquitous organelles with a neutral lipid core composed of triglycerides (TGs) and sterol esters (SEs). They are surrounded by a polar lipid monolayer composed of phospholipids (PLs), free sterols and associated proteins (perilipins, CIDE, septin, among others) anchored to the lipid monolayer (Figure 1A). LDs typically range in size from 0.1 μm to 6 μm, but in some adipocytes, they can expand to over 100 μm (Walther and Farese, 2012; Yang et al., 2012; Yu and Li, 2017).

LDs’ size and lipid composition vary significantly depending on the cell type (Figure 1B), reflecting their diverse functions and metabolic roles. In yeast, LDs are generally less than 1 μm in diameter, whereas mammalian white adipocytes can harbor a single LD that occupies the entire cytoplasm and spans hundreds of microns (Cushman, 1970; Czabany et al., 2008). This structural diversity underscores the adaptability of LDs to distinct metabolic demands and cellular environments.

#### 2.1.1 LD core composition

The lipid profile of LDs differs markedly between cell types: in yeast, TGs and SEs are present in similar amounts, in adipocytes, TGs dominate, and in macrophage foam cells, SEs predominate (Bartz et al., 2007). Beyond cell type, LDs’ lipid composition is influenced by various factors, including nutrient availability, metabolic state, enzymatic activity and pathological conditions. High nutrient availability, such as a high-calorie diet, increases TG saturation in LDs, while nutrient deprivation decreases LD hydrophobicity and increases SEs content (Ko et al., 2024; Ventura et al., 2023). A metabolic state where there is glucose deprivation reduces the TG to SE ratio, driven by TG lipolysis, and promotes transition of SEs to liquid-crystalline phase, which also impacts the lipid packing of the PL lipid layer (Rogers et al., 2022). Enzymatic activities like lipolysis, driven by enzymes such as adipose triglyceride lipase (ATGL), regulate the breakdown of TGs to free fatty acids and glycerol (Nielsen et al., 2014). Finally, pathological conditions such as obesity are associated with increased TG content in LDs (Ko et al., 2024; Wairimu, 2024). These observations underscore that LD lipid composition is not fixed, but reflects a finely tuned balance between cellular function, metabolic demands, and environmental influences.

#### 2.1.2 PL monolayer composition

The PL monolayer functions primarily to stabilize the core as an emulsion of neutral lipids within the cell. Additionally, it plays a crucial role in regulating the morphology, structure, and function of LDs (Wölk and Fedorova, 2024). The composition of this monolayer includes different types of PLs, part of phosphatidylcholine (PC), phosphatidylethanolamine (PE) and phosphatidylinositol (PI) PLs groups. Notably, the PL composition of LDs can vary markedly across cell types and tissues, reflecting specific metabolic needs and functions (Bartz et al., 2007; Fujimoto and Parton, 2011; Leber et al., 1994; Tauchi-Sato et al., 2002; Thiam et al., 2013b).

In adipocytes, LDs are characterized by an adapted PL monolayer that supports the storage of large amounts of TGs (Klemm and Carvalho, 2024). In steroidogenic cells, LDs have specific PLs that support steroid hormone synthesis (Talbott et al., 2020). In hepatic cells, LDs have PLs compositions dependent on the cell state. In quiescent cells, LDs resemble those in steroidogenic cells, while in regenerating states, the LDS resemble those in adipocytes (García-Arcos et al., 2010).

The composition of the PL monolayer plays a critical role in determining the LDs’ size and stability. Smaller LDs are composed of PLs with saturated fatty acyl chains or longer hydrocarbon tails (e.g., dipalmitoylphosphatidylcholine), allowing for tight packing due to stronger van der Waals interactions. Larger LDs tend to contain PLs with unsaturated fatty acyl chains (e.g., dioleoylphosphatidylcholine), which increase monolayer fluidity and facilitate LD expansion. Finally, not only the chains but also the headgroup of the PLs play a role in the monolayer properties. Bulky or charged headgroups tend to reduce packing density and promote larger LD size, which explains why phosphatidylethanolamine is enriched in small LDs, whereas phosphatidylcholine is more abundant in larger ones (Ben M’barek et al., 2017; Krahmer et al., 2013; Thiam et al., 2013b).

#### 2.1.3 Associated proteins

LDs are associated with various proteins important for their function (Brasaemle et al., 2009; Cho et al., 2007). Perilipins (PLINs) are a family of proteins that coat the LD surface and play key roles in LD biogenesis and lipolysis: the breakdown of TGs into free fatty acids and glycerol (Ducharme and Bickel, 2008; Hsieh et al., 2012; Rowe et al., 2016; Stribny and Schneiter, 2023). PLINs were the first proteins associated with LDs to be characterized, primarily due to their crucial metabolic role in regulating TG storage and release from LDs (Brasaemle et al., 2009; Greenberg et al., 1991).

LDs are also enriched with other proteins involved in lipid metabolism, such as acyl-CoA synthetase (ACS) and diacylglycerol acyltransferase (DGAT), which are crucial for lipid synthesis and storage (Cases et al., 1998, 2001; Kassan et al., 2013; Kuerschner et al., 2008; Poppelreuther et al., 2012; Shockey et al., 2006; Stone et al., 2009; Xu et al., 2012).

Genomic screening has identified multiple genes involved in LD formation and accumulation, influencing their number, size, and spatial distribution (Guo et al., 2008). More recently, proteomic analyses have expanded this understanding by characterizing the repertoire of proteins associated with LDs (Gianazza et al., 2025). Using proteomic analysis, Sun et al. (2023) identified more than 250 proteins in purified LDs from zebrafish liver, including unique proteins implicated in the regulation of LD dynamics.(Sun et al., 2023). In 2024, Omata et al. (2024) reported that LDs isolated from *Arabidopsis thaliana* leaves contain MYOSIN BINDING PROTEIN14 (MYOB14), a protein that may facilitate interactions between LDs and the cytoskeleton (Omata et al., 2024). A proteomic study on alcohol-associated fatty liver disease revealed alterations in the proteome, with several affected proteins linked to fatty acid synthesis, incorporation, and lipolysis (Perumal et al., 2024). Similarly, a proteomic study revealed that carnosine treatment in rats, a natural dipeptide known for its protective effects against oxidative stress in nonalcoholic fatty liver disease (NAFLD), led to the downregulation of PLIN2 and ApoE (Moreto et al., 2024).

Advanced techniques such as proximity labeling proteomics using APEX2, an engineered ascorbate peroxidase used for proximity labeling in proteomics, have been developed to accurately identify LD proteomes with a rapid and spatially-resolved mapping of protein interaction and subcellular proteomes in living cells (Bersuker et al., 2018; Peterson et al., 2021). Recent advances have enabled the systematic identification of monolayer-integrated proteins. For instance, studies have shown that DHRS3 contains an amphipathic α-helix, which plays a crucial role in its integration into the LD monolayer and the cytoplasmic leaflet of the ER membrane (Pataki et al., 2018).

The protein composition of LDs can vary substantially depending on their cellular origin, metabolic state, and function. Proteomic analyses have shown that most LDs are associated with over 100 distinct proteins, encompassing structural proteins, lipid metabolism enzymes, membrane trafficking regulators, and signaling molecules (Krahmer et al., 2013). Globally, the protein composition can vary depending on the cell type and metabolic state, reflecting the LD’s role in cellular processes like inflammation and immune responses (Walther and Farese, 2012; Zadoorian et al., 2023).

The size and lipid composition are other factors that could also favor the LD protein composition (Roberts and Olzmann, 2020). This variation in protein LDs content is important to adapt to different cellular needs, playing a role in various cellular processes (Henne et al., 2018; Zadoorian et al., 2023). In mice fed a high-fat diet, LD enlargement has been linked to the sequestration of proteins from other organelles, a process that can impair the normal function of these organelles (Krahmer et al., 2018). Moreover, mutations in proteins such as ABHD5 and PNPLA3, important for TG hydrolysis and storage, can dysregulate lipid metabolism, leading to conditions similar to fatty liver disease (Teskey et al., 2024).

### 2.2 Biogenesis of LDs

**Figure 2:**
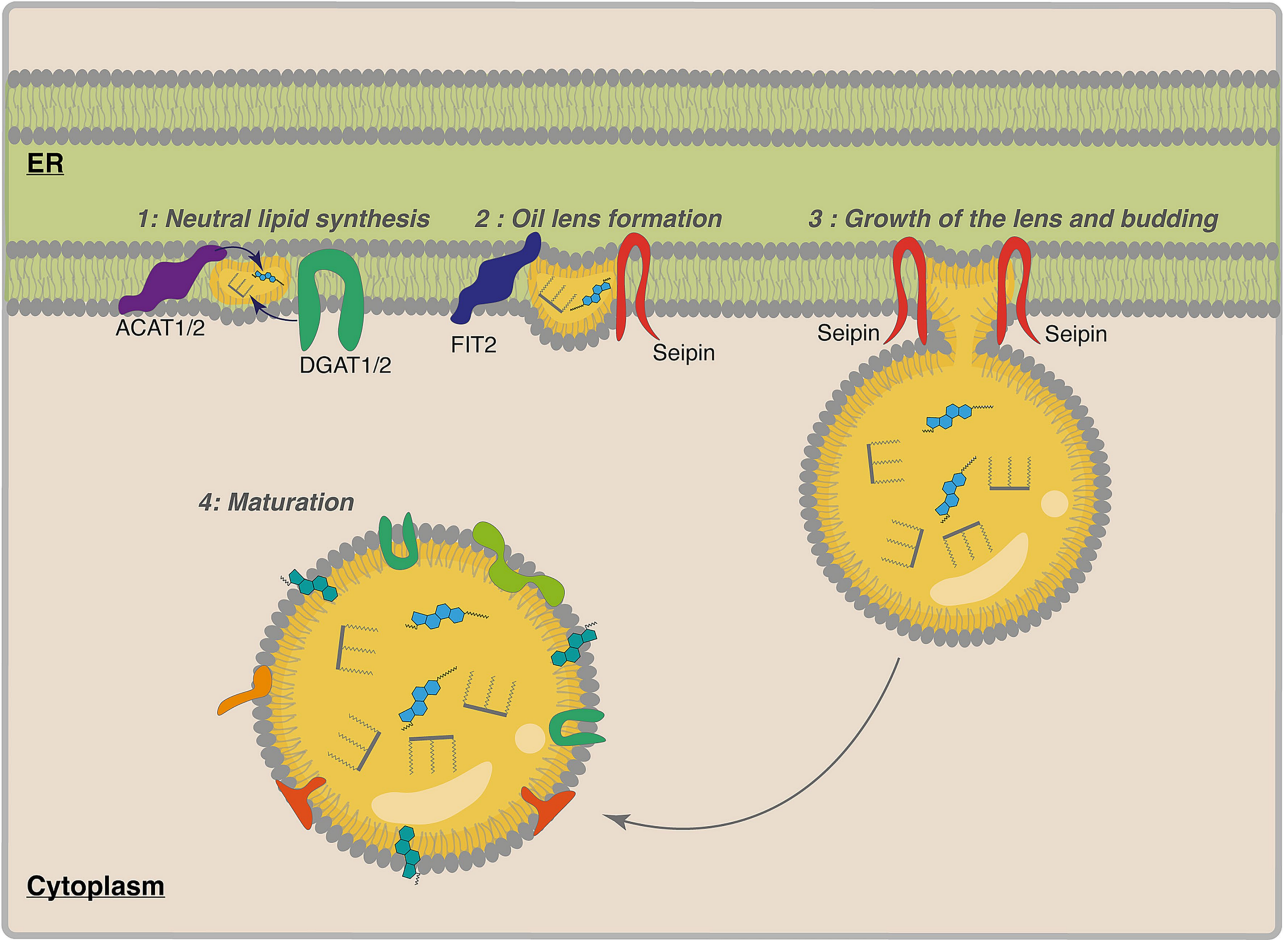
LD biogenesis, key steps and proteins involved: schematic representation illustrating the sequential stages of LD formation, highlighting the roles of specific proteins at each step. (1) Neutral lipid synthesis: Enzymes such as DGAT1/2 and ACAT1/2 catalyze the formation of triglycerides and sterol esters in the endoplasmic reticulum (ER) membrane; (2) Oil lens formation: FIT2 and Seipin proteins facilitate the coalescence of neutral lipids into a lens between the ER bilayer leaflets; (3) Growth and budding: Seipin oligomers stabilize the ER-LD contact site, coordinating lipid transfer and preventing premature scission as the lens grows and buds from the ER; (4) Maturation: The nascent LD emerges into the cytoplasm, surrounded by a phospholipid monolayer derived from the ER, forming a mature LD.

The biogenesis of LDs is a complex and not yet fully understood process, involving multiple steps and regulatory factors. While *de novo* formation is the most well-characterized pathway, fission also plays a crucial role in LD formation (Buhman et al., 2001; Jacquier et al., 2011, 2013; Long et al., 2012). LDs *de novo* formation begins in the ER, where enzymes responsible for synthesizing neutral lipids are localized (Buhman et al., 2001; Goodman et al., 1964; Walther et al., 2017). LD biogenesis occurs through several distinct steps: (1) neutral lipid synthesis and accumulation in the ER bilayer, (2) formation of an oil lens within the ER membrane, (3) growth of the lens and budding from the ER, and (4) maturation of the LD in the cytoplasm (Figure 2).

The synthesis of TGs and SEs is one of the earliest and best-characterized steps in LD biogenesis, occurring primarily in the ER. The process involves several key steps and different enzymes. First, free fatty acids are activated by Acyl coenzyme A (CoA) and synthetase (ACS) enzymes, which esterify fatty acids to CoA esters. Next, these activated fatty acids are used as substrates to synthesize neutral lipids, which accumulate between the leaflets of the ER bilayer (Wilfling et al., 2014a).

For the synthesis of TGs, DGAT1 and DGAT2 catalyze the synthesis of TGs by combining fatty acyl-CoA with diacylglycerol (Cases et al., 1998, 2001; Routaboul et al., 1999; Yen et al., 2008). The two DGATs localized to the ER are crucial for the final steps of TGs synthesis, making them key targets for modifying LD formation (Cheng et al., 2020; Inloes et al., 2014; Stone et al., 2009; Yang et al., 2020).

In parallel to the formation of TGs, SEs formation is catalyzed by another family of enzymes, sterol O-acyltransferase (SOAT), also known as acyl-CoA cholesterol acyltransferase (ACAT). Two members of this family, ACAT1 and ACAT2 are essential for cholesterol esterification, thereby promoting the accumulation of SEs in nascent LDs (Chang et al., 2009; Liu et al., 2005; Rogers et al., 2015).

As neutral lipids accumulate, they form a lipid lens within the ER membrane. Once this lens reaches a critical size and specific lipid composition, it buds off from the ER to form an LD (Khandelia et al., 2010; Long et al., 2012; Soni et al., 2009; Thiam et al., 2013b; Wang et al., 2014; Wilfling et al., 2013). This process is regulated by fat-storage-inducing transmembrane (FIT) proteins, particularly FIT2, which facilitate the partitioning of neutral lipids within the ER, ensuring proper lens formation and subsequent budding (Becuwe et al., 2020; Graff and Schneiter, 2024; Gross et al., 2010; Miranda et al., 2014).

Another important protein for LD biogenesis is Seipin, a transmembrane protein found at the junctions between the ER and cytosolic LDs (Binns et al., 2010; Fei et al., 2011; Szymanski et al., 2007). This protein is involved in converting nascent to mature LDs by enabling the transfer of lipids from the ER to the nascent LDs (Wang et al., 2016). Seipin, therefore, contributes to TG nucleation and the budding of LDs (Chung et al., 2019; Kim et al., 2022). PLIN proteins such as PLIN-3 have also been suggested to be important regulators of LD formation, due to their attraction to ER membranes enriched with diacylglycerol; it could play a role in the formation of LDs (Brasaemle et al., 2009; Greenberg et al., 1991; Hsieh et al., 2012; Khaddaj et al., 2023; Rowe et al., 2016; Skinner et al., 2009; Stribny and Schneiter, 2023).

Furthermore, after budding from the ER, LDs can continue to grow through different mechanisms, contributing to their overall expansion. The increase in the size of LDs could be due to two different mechanisms: LD growth or LD fusion. LD growth occurs at the surface of LDs; this requires the trafficking of enzymes for the local synthesis of TGs at the surface of LDs (Wilfling et al., 2013; Wilfling et al., 2014b). Certain ER proteins, such as atlastin, regulate LD size (Klemm et al., 2013), while others facilitate the physical tethering of LDs to the ER. Another example is the COP1 protein, which removes PLs at the LDs surface, increasing the surface tension and facilitating the fusion of LDs with other membranes (Wilfling et al., 2013). Once the ER-LD connection is established, triglyceride synthesis enzymes relocate from the ER to LDs, promoting their expansion (Poppelreuther et al., 2012; Stone et al., 2009; Thiam et al., 2013a; Wilfling et al., 2013; Wilfling et al., 2014b).

In parallel to core expansion, the PL surface expansion is also crucial. Synthesis of PC is needed for the LD expansion. This synthesis is catalyzed by different enzymes such as cytidylyltransferase (CCT) and choline-phosphotransferase (CPT) (Krahmer et al., 2011). The fusion of LDs is also possible, through direct coalescence or by a ripening process called permeation. Direct coalescence involves the physical merging of two LDs when their monolayer surfaces come into contact, resulting in a larger droplet. Permeation refers to the diffusion of triglycerides from a smaller, less stable LD to a larger one (Thiam et al., 2013b; Xu N. et al., 2024). This process is mediated by specific proteins from the cell death-inducing DFF45-like effector (CIDE) family (Gong et al., 2011; Lyu et al., 2021; Murphy et al., 2010; Sun et al., 2013; Xu L. et al., 2024).

### 2.3 LD functions

**Figure 3:**
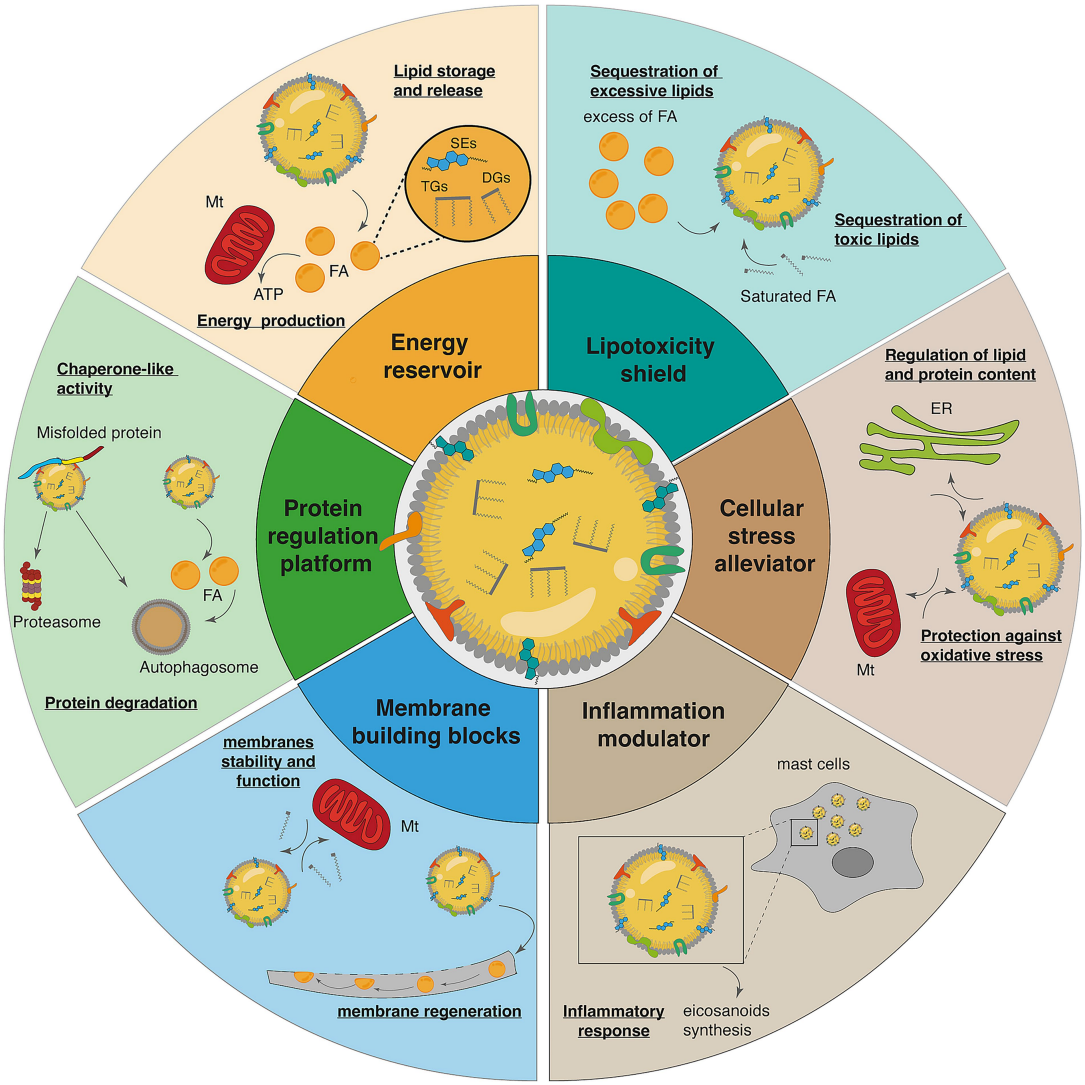
LD functions. *Energy reservoir*: LDs store neutral lipids and cholesterol, serving as energy reservoirs for cellular needs. *Lipotoxicity shield*: LDs protect cells from lipo-toxicity by sequestering potentially toxic lipids into their neutral lipid core. *Cellular stress alleviator*: LDs play a crucial role in mitigating various cellular stresses. They help reduce lipotoxic and ER stress by regulating lipid and protein content within the ER. Additionally, LDs protect against oxidative stress by interacting with mitochondria, contributing to cellular homeostasis and survival. *Inflammation modulator*: LDs are involved in inflammatory responses through the synthesis and metabolism of eicosanoids, one example is in immune cells, including mast cells. *Membrane building blocks*: LDs act as reservoirs for cholesterol and acyl-glycerols needed for membrane formation and maintenance. *Protein regulation platform*: LDs serve as platforms for protein binding and degradation, contributing to cellular protein homeostasis. They exhibit chaperone-like activity, aiding in the management of misfolded proteins, while also facilitating protein degradation through interactions with the proteasome and autophagosome. SEs, sterol esters; DGs, diglycerides; TGs, triglycerides; FA, fatty acids; ATP, adenosine triphosphate; ER, Endothelial reticulum; Mt., Mitochondria (the figure is adapted from Petan et al., 2018).

Following their formation and maturation, LDs serve a range of functions crucial for maintaining cellular balance, including lipid storage, membrane biosynthesis, and protein regulation (Figure 3 and Table 1).

**Table 1:** Summary of the main LD functions.

Function	Details	References
Energy reservoir	LDs store neutral lipids and cholesterol, serving as energy reservoirs for cellular needs	Barbosa et al. (2015); Bickel et al. (2009); Brasaemle (2007); Ducharme and Bickel (2008); Guo et al. (2008); Walther & Farese (2012)
Lipotoxicity shield	LDs protect cells from lipotoxicity by sequestering potentially toxic lipids into their neutral lipid core	Herms et al. (2013); Listenberger et al. (2003); Nguyen et al. (2017); Obaseki et al. (2024); Plötz et al. (2016)
Cellular stress alleviator	LDs play a crucial role in relieving various cellular stresses, including oxidative stress and ER stress	Bensaad et al. (2014); Chen et al. (2017); Chitraju et al. (2017); Kuramoto et al. (2012); Mukhopadhyay et al. (2017); Ploegh (2007); Puskás et al. (2010); Velázquez et al. (2016); Vevea et al. (2015)
Membrane building blocks	LDs act as reservoirs for cholesterol and acyl-glycerol needed for membrane formation and maintenance	Gouna et al. (2021); Mou et al. (2020); Ozsvár et al. (2018); Plewes et al. (2020)
Inflammation regulator	LDs are involved in inflammatory responses through the synthesis and metabolism of eicosanoids	Bozza and Viola (2010); Cruz et al. (2020); Zhang et al. (2021)
Protein regulation platform	LDs serve as platforms for protein binding and degradation, contributing to cellular protein homeostasis	Chen et al. (2017); Henne et al. (2018); Moldavski et al. (2015); Ohsaki et al. (2006); Roberts and Olzmann (2020); Shpilka et al. (2015); Velázquez et al. (2016)

By storing neutral lipids, LDs fulfill their primary function as energy reservoirs. The stored TGs and SEs are mobilized in response to energy demands, making LDs essential for maintaining cellular energy homeostasis (Barbosa et al., 2015; Bickel et al., 2009; Brasaemle, 2007; Ducharme and Bickel, 2008; Guo et al., 2008; Walther and Farese, 2012). In this function, PLIN proteins play an important role in the stabilization of the LDs and regulation of lipid storage (Bickel et al., 2009; Kimmel and Sztalryd, 2016). The nutrient status of cells is important for LD formation and accumulation. In conditions of high availability of exogenous lipids, but also nutrient-deprivation conditions, the formation of lipid droplets is stimulated (Cao et al., 2019; Hariri et al., 2018; Krahmer et al., 2011; Kwon et al., 2017; Rambold et al., 2015; Seo et al., 2017; Soayfane et al., 2016). Interestingly, in starvation conditions, the cells switch their energy source from glucose to high-energy fatty acids to stimulate ATP production via mitochondrial fatty acid catabolism (Gerhart-Hines et al., 2007). The autophagic breakdown of membranes provides LDs with fatty acids, which are subsequently catabolized in the mitochondria (Rambold et al., 2015).

Excessive accumulation of fatty acids, particularly long-chain saturated fatty acids, could lead to lipotoxicity. LDs play an important role in mitigating this toxicity by sequestrating these fatty acids into their neutral core (Herms et al., 2013; Listenberger et al., 2003; Nguyen et al., 2017; Obaseki et al., 2024; Plötz et al., 2016).

In addition to their protective role against lipotoxicity, LDs contribute to cellular homeostasis under stress conditions. They play a critical role in the cellular stress response, helping to mitigate oxidative stress, ER stress, and other stressors. LD integrity is equally important; for instance, PLIN5-deficient mice exhibit elevated ROS levels (Kuramoto et al., 2012). Moreover, inhibition of LD formation has been linked to diminished cellular protection against ROS toxicity, resulting in reduced survival under hypoxia–reoxygenation conditions (Bensaad et al., 2014). Conversely, cells exposed to oxidative stress show an increased accumulation of LDs. Cells subjected to oxidative stress display a marked increase in LD accumulation, a response thought to sequester and store peroxidized lipids, thereby limiting membrane damage and preserving cellular integrity (Ackerman et al., 2018; Bailey et al., 2015; Bensaad et al., 2014; Schlaepfer et al., 2015).

LDs play an important protective role against ER stress by regulating ER lipid and protein homeostasis (Chen et al., 2017; Chitraju et al., 2017; Mukhopadhyay et al., 2017; Ploegh, 2007; Puskás et al., 2010; Velázquez et al., 2016; Vevea et al., 2015). Therefore, disruption in LD turnover could lead to ER stress and be associated with LD accumulation (Bosma et al., 2014; Chitraju et al., 2017; Fei et al., 2009; Fuchs et al., 2012; Lee et al., 2012; Mukhopadhyay et al., 2017; Velázquez et al., 2016).

Maintaining cellular protein homeostasis relies on proper protein turnover. LDs play an important function in protein quality control (Roberts and Olzmann, 2020). By sequestering toxic proteins that accumulate during metabolic stress, LDs contribute to the unfolded protein response (UPR), a process regulated by PLIN2 (Chen et al., 2017, p. 20). Another role of LDs in the context of protein quality control is to act as chaperones by preventing protein misfolding (Henne et al., 2018). They also serve as a platform for proteasomal autophagic degradation, the main protein degradation pathways (Moldavski et al., 2015; Ohsaki et al., 2006). It has been shown that Lpl1, a phospholipase part of the LD, is required for efficient proteasomal protein degradation (Weisshaar et al., 2017). LDs also support autophagosome formation by donating lipids, thereby facilitating the autophagic process (Shpilka et al., 2015; Velázquez et al., 2016).

LDs play a crucial role in regulating inflammation through several mechanisms. LDs serve as a platform for the synthesis and regulation of eicosanoids, which are lipid-based signaling molecules, acting as important inflammatory mediators. LDs compartmentalize enzymes and substrates required for eicosanoid production, offering a rapid mobilization of eicosanoid precursors during inflammatory responses (Bozza and Viola, 2010; Cruz et al., 2020). They also regulate the function of various immune cells, such as mast cells, where LDs act as sites for lipid mediator synthesis, thereby influencing the release of inflammatory mediators (Zhang et al., 2021).

LDs also contribute to membrane formation and maintenance by serving as lipid reservoirs. The construction or repair of membranes relies on the availability of lipids, which LDs supply. By storing cholesterol as cholesterol esters, LDs can hydrolyze them to release free cholesterol, ensuring mitochondrial function and overall cellular membrane stability (Plewes et al., 2020). Furthermore, LDs store lipids needed for the regeneration of membranes as well as processes such as remyelination of neurons (Gouna et al., 2021; Mou et al., 2020; Ozsvár et al., 2018).

### 2.4 LDs in brain physiology and neurodegeneration

**Figure 4:**
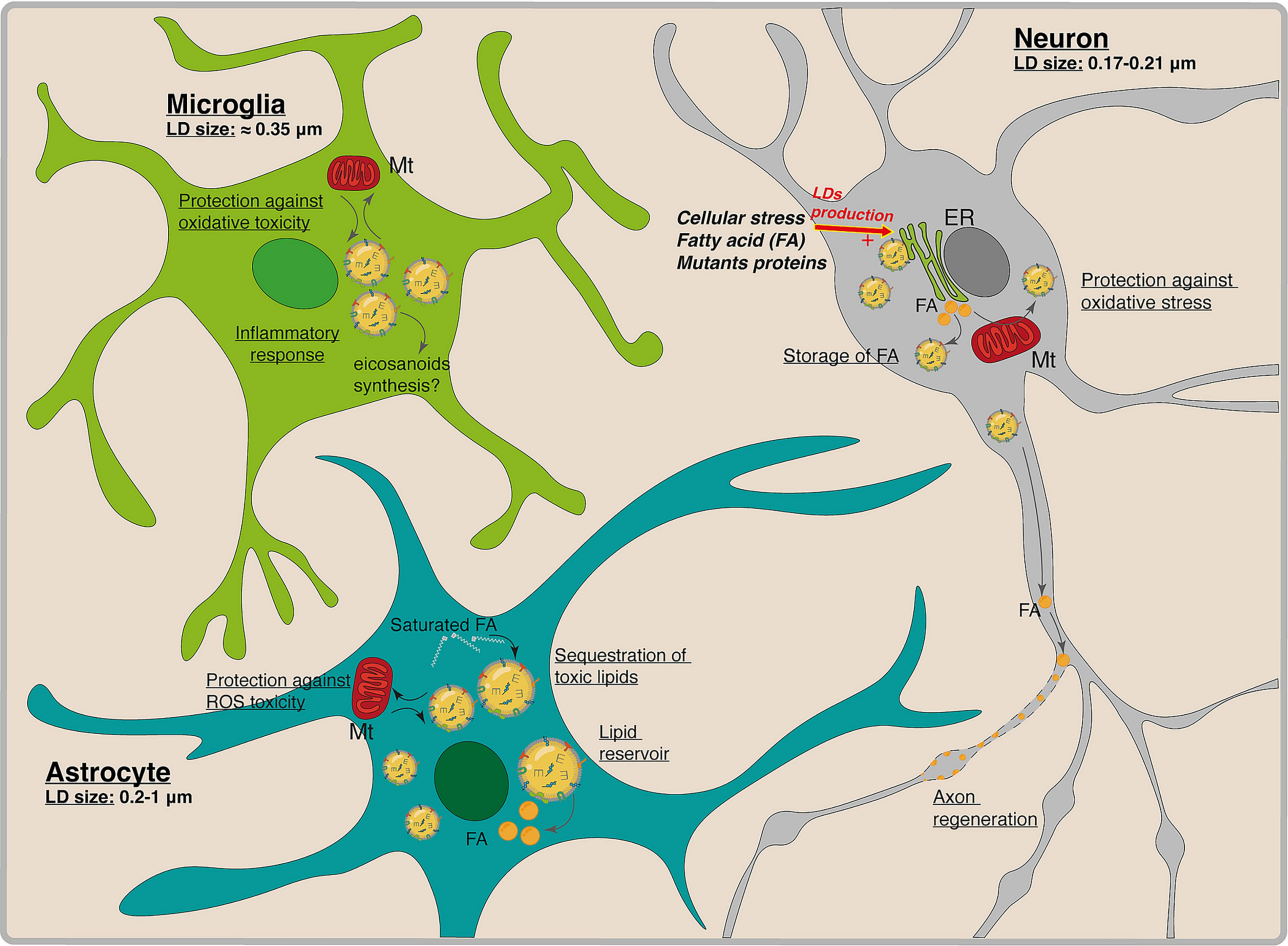
LDs in the brain and their potential functions. Schematic representation of LDs size and functions in neurons, microglia and astrocytes. Across these three brain cell types, LDs play various roles: Storage of fatty acids (FA); protection against cellular toxicity, lipid reservoir, sequestration of toxic lipids and inflammatory response. In physiological conditions, neurons do not necessarily produce LDs. However, the LDs production is stimulated by cellular stress, incubation with FA or the presence of mutant proteins. ER, Endothelial reticulum; Mt., Mitochondria.

LDs in the brain possess the same fundamental structure as those in other tissues, consisting of a hydrophobic core of neutral lipids encased by a phospholipid monolayer with associated proteins. They are present in various brain cell types, such as neurons, astrocytes, microglia, oligodendrocytes and ependymal cells (Ralhan et al., 2021) (Figure 4). While their fundamental structure is conserved, brain LDs can harbor distinct protein profiles that vary by cell type. For instance, PLIN2 is broadly expressed in the brain’s gray matter, particularly in neurons of the cortex, hippocampus, and cerebellum. In contrast, PLIN3 is predominantly found in astrocytes and white matter, while PLIN5 is mainly present in white matter (Conte et al., 2022). Moreover, overall lipid distribution varies markedly among brain cell types (Fitzner et al., 2020), and some studies have also reported differences in LD lipid composition.

The presence of LDs in neurons has so far been demonstrated only *in vitro* or under specific conditions such as aging, pathological states, or development. In neurons, LD formation is more restricted compared to other cell types. However, recent work by Manceau et al. (2024) has identified neuronal LDs both *in vivo* and *in vitro* across two species, Drosophila (fly) and mouse. In adult flies, LD spatial distribution is comparable between males and females, with the highest concentrations located in brain regions associated with learning, vision, and the homeostatic regulation of sleep and body fat (e.g., mushroom body, optic lobes). In mouse hypothalamic neurons (N46 and GT1-7 cell lines), LDs were present in 33% of GT1-7 neurons and 13% of N46 neurons. Oleate (C18:1) and palmitate (C16:0) were the most abundant fatty acids esterified in neuronal LDs, alongside smaller amounts of palmitoleate and stearate. *In vivo* analysis of adult mouse brain sections under physiological conditions confirmed LD presence in 8%–12% of arcuate nucleus neurons. Together, these findings from cell lines and intact tissue indicate that hypothalamic neurons in both flies and mammals actively esterify fatty acids into LDs, pointing to conserved mechanisms of lipid storage in neuronal populations regulating energy balance (Manceau et al., 2024). This study also revealed the difficulty in identifying neural LDs in physiological conditions due to their small size and low prevalence.

Another study detected LDs in hippocampal sections from adult control mice, present in both neurons and microglia. Neuronal LDs were distributed in specific regions of the hippocampus, with notable presence in the cornu ammonis (CA1) and CA3 areas, while being less abundant in dentate gyrus neurons. Furthermore, CA1 neurons had smaller LDs (~174 nm) with lower density (56% less) compared to CA3 neurons, where the LDs are approximately 211 nm (Seferi et al., 2024).

Using the tdTom-Plin2 reporter mouse, researchers observed LDs in various brain regions, including the olfactory bulb, cortex, lateral ventricles, and cerebellum. Molecular analysis confirmed that tdTom-Plin2 expression did not alter lipid composition. Cell-type analysis revealed LDs in neurons (~13%), astrocytes (~40%), ependymal cells, microglia, endothelial cells, and oligodendrocytes. Traditional lipid staining methods failed to detect LDs as effectively as the tdTom-Plin2 model, highlighting the need for improved detection techniques (Madsen et al., 2024; Petrelli et al., 2024).

Astrocytes, the primary metabolic supporters of neurons, exhibit a high propensity for LD formation, with isolated cortical astrocytes showing a 35% LD prevalence (0.2–1 μm in size) under basal conditions (Smolič et al., 2021a; Smolič et al., 2021b). Under stress, such as neuronal oxidative injury or neuroinflammatory stimuli, astrocytes rapidly accumulate LDs through apolipoprotein-mediated lipid transfer (Ioannou et al., 2019; Kwon et al., 2017). These LDs serve as neuroprotective reservoirs, sequestering peroxidized lipids and free fatty acids to mitigate ROS and prevent lipotoxicity (Cheng et al., 2020; Olzmann and Carvalho, 2019). For example, in Alzheimer’s disease (AD) models, astrocytes take up oxidized lipids released by stressed neurons and esterify them into LDs via acyl-CoA synthetase (ACSL4), thereby protecting neurons from lipid-induced damage (Goodman et al., 2024; Moulton et al., 2021).

Microglia, the brain’s immune sentinels, accumulate LDs in response to aging and inflammatory stimuli, but their distribution and size differ substantially from those in astrocytes. In the hippocampus, microglial LDs average 350–386 nm in diameter, forming sparsely but consistently in regions like the CA1 and CA3 subfields (Manceau et al., 2024). Aging drives microglial LD accumulation via TLR4-NF-κB signaling, triggering a pro-inflammatory transition characterized by IL-1β and TNF-α release (Marschallinger et al., 2020). These lipid-laden microglia exhibit impaired phagocytosis and heightened oxidative stress, exacerbating neuronal damage in neurodegenerative diseases (Claes et al., 2021; Robb et al., 2025).

During brain development, LDs play an important role by protecting stem cells from oxidative stress. For instance, inhibiting LD formation in glial cells has been shown to reduce their proliferative capacity while also increasing lipid peroxidation (Bailey et al., 2015; Morrison et al., 2000; Ralhan et al., 2021). LDs play a crucial role in immune responses and in protecting organelles from damage (Zhang Y. et al., 2025). In the aging brain, LD accumulation has been observed across various cell types, with microglia being the most affected (Ralhan et al., 2021). Notably, microglia containing LDs exhibit altered functionality, including increased oxidative stress, heightened proinflammatory signaling, and reduced phagocytic capacity (Marschallinger et al., 2020; Shimabukuro et al., 2016).

The role of LDs as reservoirs in neurons was recently demonstrated in the work of Kumar et al. (2025). They revealed that neurons store TG in LDs, serving as an energetic reserve for synaptic activity, with the enzyme DDHD2 as the principal TG lipase. Moreover, mice lacking this enzyme accumulate LDs in the presynaptic compartment, leading to impaired synaptic function, reduced neurotransmitter release and cognitive deficits. This finding suggests that defects in LD metabolism, such as the DDHD2 mutation, may contribute to neurodegenerative diseases by depriving synapses of necessary energy (Kumar et al., 2025).

LDs may play a crucial role in various neurodegenerative diseases, serving as both indicators and potential contributors to disease progression. In aging and neurodegenerative conditions, there is a notable increase in LD accumulation within various brain cells, including neurons, microglia, and astrocytes (Farmer et al., 2020). This accumulation is often accompanied by changes in LD protein composition, such as increased expression of PLIN2 as observed in Alzheimer’s disease (Conte et al., 2022). LD dysregulation can lead to cellular dysfunction, metabolic abnormalities, and exacerbation of neurodegenerative processes (Farmer et al., 2020; Zhang Y. et al., 2025).

AD is the most common form of dementia. Alois Alzheimer originally described three key neuropathological features: neurofibrillary changes, amyloid deposits, and glial lipid accumulation. While tau and amyloid have been extensively studied, LD accumulation in AD has been largely overlooked until recently (Farmer et al., 2020). Recent studies revealed that increased LD formation in AD brains correlates with neurogenesis defects and inflammatory responses. This was shown by the accumulation of LDs in the subventricular zone of AD models, along with evidence that cholesterol esters (CEs) within LDs contribute to tau pathology by impairing proteasome activity (Hamilton et al., 2015; Van Der Kant et al., 2019). Moreover, apolipoproteins, particularly ApoE, play a key role in lipid transport. The APOE4 allele, the strongest genetic risk factor for late-onset AD, is associated with inefficient lipid shuttling and increased LD accumulation in astrocytes (Farmer et al., 2019).

LDs are also involved in managing oxidative stress, with astrocytes utilizing LDs to store peroxidized lipids from hyperactive neurons. This protective mechanism, known as the neuron-astrocyte metabolic coupling model, allows neurons under stress to export oxidized lipids to astrocytes as a means of neuroprotection. For example, a study found that elevating reactive oxygen species (ROS) levels in neurons causes an SREBP-dependent production of peroxidized lipids, which are then exported to glial cells and integrated into LDs (Goodman et al., 2024). However, this protective mechanism may be compromised in neurodegenerative conditions, leading to increased cellular damage (Farmer et al., 2020; Goodman et al., 2024).

In amyotrophic lateral sclerosis (ALS), alterations in lipid metabolism and LD dynamics have been observed, suggesting a potential role in disease pathogenesis. The accumulation of LDs in glial cells, particularly in astrocytes, where the number is increased by 3-fold in the presence of TDP-43 cytoplasmic inclusions (Velebit et al., 2020).

Moreover, in Huntington’s disease (HD), a mouse model expressing mutant huntingtin protein shows increased LD accumulation in primary striatal neurons and glia, associated with defects in the autophagic pathway (Martinez-Vicente et al., 2010).

The complex interplay between LDs, cellular metabolism, and inflammatory responses underscores their significance in brain health and disease progression across multiple neurodegenerative disorders (Farmer et al., 2020; Smolič et al., 2021b; Zhang L. et al., 2025). In summary, alterations in LD dynamics, particularly in aging and neurodegenerative diseases, can lead to impaired cellular function, increased oxidative damage, and disrupted inflammatory responses, highlighting their complex yet vital role in brain health and disease.

## 3 LDs interaction with α-Syn and their implication in PD

Among neurodegenerative diseases, PD presents a particularly intriguing connection to LDs. Emerging evidence suggests that LDs may interact with key pathological features of PD, including mitochondrial dysfunction, lipid dysregulation, and the aggregation of α-Syn (Alecu and Bennett, 2019; Fanning et al., 2020; Flores-Leon and Outeiro, 2023; Galper et al., 2022; Girard et al., 2021; Han et al., 2018; Li et al., 2019). LDs may influence these processes by serving as reservoirs for lipids that can either protect against toxicity or contribute to disease progression, depending on cellular conditions. Notably, many studies have highlighted a direct interaction between LDs and α-Syn. This interaction opens questions about the role of LDs in α-Syn aggregation, clearance, and toxicity, highlighting a potential mechanistic link between lipid metabolism and neurodegeneration.

### 3.1 α-Syn and binding to lipids

α-Syn is a small (14 kDa) protein abundantly expressed in neurons, astrocytes (di Domenico et al., 2019), peripheral tissues, and blood (Malek et al., 2014; Witt, 2013). Its precise physiological function, however, remains unclear, and its structure can vary depending on cellular location and environmental conditions (Lehtonen et al., 2019).

**Figure 5:**
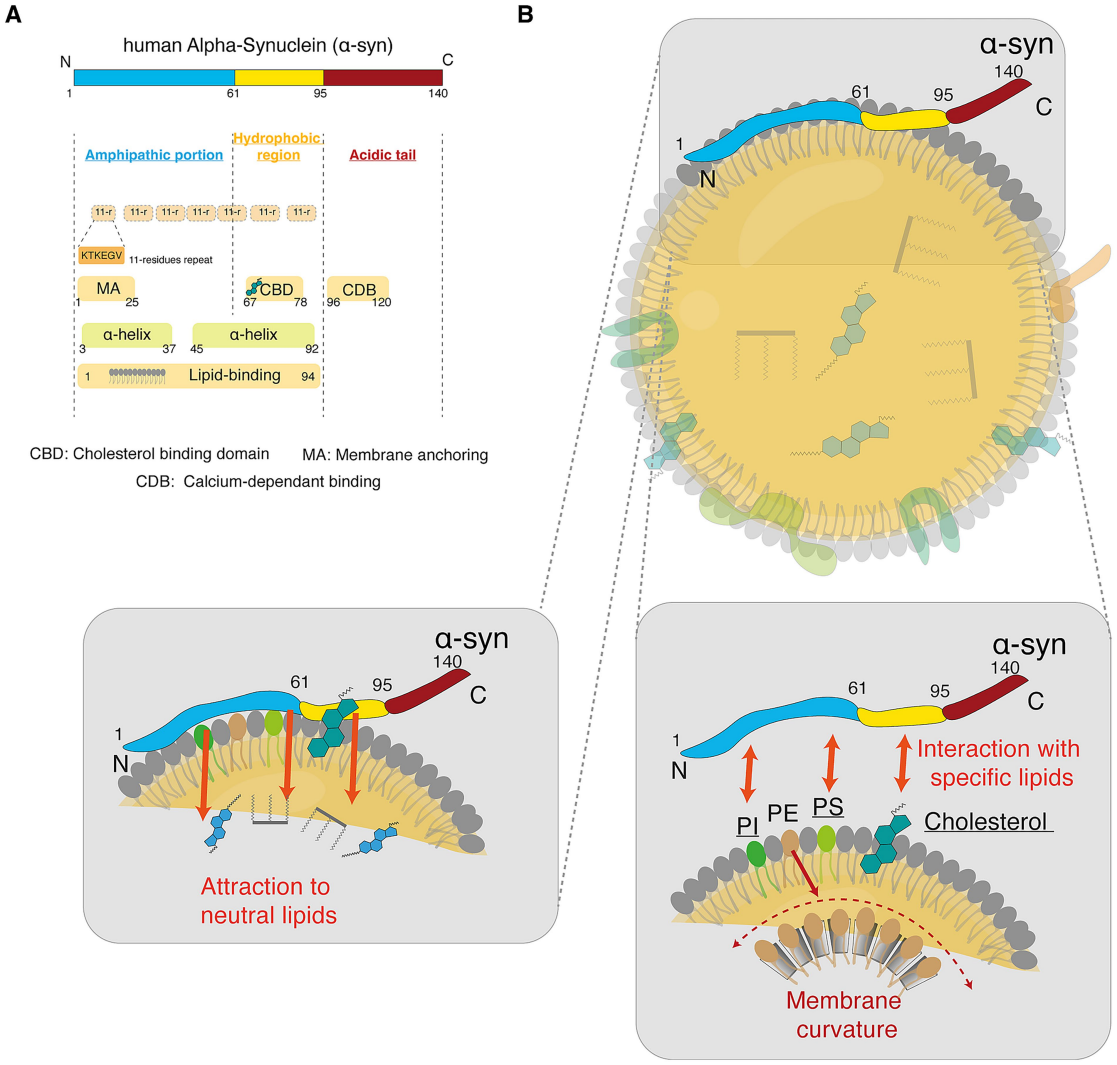
α-Syn, lipids and LD interaction. **(A)** Schematic representation of α-Syn primary structure, with its three distinct domains. Several regions of alpha-synuclein have specific interactions with lipids. **(B)** Schematic representation of the potential interaction mechanisms (attraction vs. interaction) between α-Syn and LDs. Left panel: The LD monolayer could favorize attraction of α-Syn to the core neutral lipids, therefore favorizing the docking of α-Syn on LD membranes. Right panel: Syn has been shown to. Interaction with specific phospholipids, such as PS and PI but also with cholesterol. PS, phosphatidylserine; PE, phosphatidylethanolamine; PI, phosphatidylinositol.

The primary structure of α-Syn is constituted of distinct domains (Figure 5A), the amphipathic N-terminal portion (residues 1–60) primarily responsible for membrane binding, the hydrophobic region (previously known as the non-amyloid *β*-component (NAC) domain) (residues 61–95) important for membrane binding and aggregation, and finally a C-terminal acidic tail (residues 96–140) involved in different functions such interaction with other proteins, regulation of protein solubility, calcium binding and aggregation regulation (Bartels et al., 2010; Bisaglia et al., 2006; Bisi et al., 2021; Goedert et al., 2024; Liu et al., 2021; Runfola et al., 2020). The first 94 amino acids play a crucial role in making α-Syn prone to interacting with various lipid membranes. They can form α-helices and transition from a continuous helix to two separate helices, spanning residues 1–37 and 45–94, when the lipid membrane exhibits higher curvature (Bell and Vendruscolo, 2021; Chandra et al., 2003; Georgieva et al., 2010).

Interactions between α-Syn and lipid membranes play a critical role in PD pathogenesis (Alecu and Bennett, 2019; Battis et al., 2023; Fanning et al., 2020; Flores-Leon and Outeiro, 2023; Shahmoradian et al., 2019). In familial forms of PD, mutations often occur within the N-terminal domain of α-Syn, a region essential for lipid binding (Battis et al., 2023; Galvagnion, 2017; Gilmozzi et al., 2020; Kaur and Lee, 2020). The association of α-Syn with LDs is highly dynamic and modulated by structural features such as amphipathic helices and specific amino acid residues (Braun et al., 2017; Chorlay and Thiam, 2020). These interactions are not uniform; rather, they vary depending on factors such as lipid composition, membrane curvature, and α-Syn’s conformational state (Amos et al., 2021; Fakhree et al., 2018; Hannestad et al., 2020; Kaur and Lee, 2020; Navarro-Paya et al., 2022).

The positively charged N-terminal region contains four of the seven imperfect 11-residue repeats, with a highly conserved hexameric sequence (KTKEGV). These repeats are crucial for α-Syn interaction with membranes, as they contribute to the formation of amphipathic α-helices when α-Syn binds to membranes (Brontesi et al., 2023; Bussell and Eliezer, 2003; Croke et al., 2011; Sode et al., 2006). Specific residues in the N-terminal portion play key roles in the interaction with lipids. Residues 1–25 of the N-terminal region form a strong membrane anchor, interacting with negatively charged lipids such as POPG (1-Palmitoyl-2-oleoyl-sn-glycero-3-phosphoglycerol), PS (phosphatidylserine), and PA (phosphatidic acid) by forming an amphipathic α-helix (Fusco et al., 2014). Residues 1–12 within this region are partially embedded into the hydrophobic core of the lipid bilayer, playing a key role in anchoring α-Syn to the membrane surface. This partial insertion of the initial 12 residues into the hydrophobic chains of the lipid bilayer allows α-Syn to tightly bind lipid vesicles while maintaining a rapid equilibrium between membrane-bound and unbound states (Fusco et al., 2016; Jao et al., 2004).

As mentioned above, the N-terminal region contains four 11-residue repeats, the first three contributing to the first α-helix (residues 3–37) (Das and Mattaparthi, 2023; Sung and Eliezer, 2018). The last 11-residue repeats of this region, in addition to the three 11-residue repeats of the hydrophobic region, constitute the second α-helix (45–92) (Bisaglia et al., 2006).

The central region (residues 61–95), which encompasses the hydrophobic region, is a hydrophobic segment crucial for membrane binding and aggregation. Within this region, residues 67–78 form a distinct cholesterol-binding domain (Fantini et al., 2011). In contrast, the C-terminal region (residues 96–140) exhibits generally weak membrane interactions and remains largely disordered. Specifically, residues 96–120 display weak, calcium-dependent membrane association, while residues 121–140 contribute minimally or not at all to direct membrane interactions (Ahn et al., 2006; Clayton and George, 1998; Kiechle et al., 2020; Sarchione et al., 2021).

Given the defined lipid-binding properties of α-Syn, particularly within its N-terminal and hydrophobic region, the composition of the LD monolayer becomes highly relevant (Figure 5B). This monolayer is primarily composed of PC, PE, and PI, but can also contain other phospholipids, including lysophosphatidylcholine (LPC), phosphatidylserine (PS), and sphingomyelin (SM) (Wölk and Fedorova, 2024).

PC, the most abundant phospholipid on the LD surface, interacts only weakly with α-Syn due to its zwitterionic nature. However, the presence of anionic lipids such as PS or PG in the membrane enhances α-Syn binding (Rhoades et al., 2006; Stöckl et al., 2008). Notably, studies using PC-based nanodiscs have shown that cholesterol can further promote α-Syn–lipid interactions by modulating the hydrophobic region, thereby increasing α-Syn binding to the membrane (Jakubec et al., 2021).

PE, like PC, is a zwitterionic lipid, but it has a smaller headgroup, which can affect membrane curvature and potentially influence α-Syn binding (Jakubec et al., 2021). Indeed, a decrease in the PC: PE ratio has been shown to enhance the binding of proteins with amphipathic alpha helices (e.g., PLIN2), such as α-Syn, to lipid droplets. This suggests that PE plays a role in facilitating the association of α-Syn with lipid droplets by altering the surface properties of the droplets (Jakubec et al., 2021; Kaur and Lee, 2021; Listenberger et al., 2018). Interestingly, a recent study suggests that α-Syn binding is driven by membrane packing defects rather than anionic charge. As a zwitterionic lipid, PE contributes to packing defects that are essential for α-Syn binding. Studies indicate that membranes with higher phospholipid unsaturation, which generate more packing defects, enhance α-Syn binding (Johnson et al., 2025).

Due to its negative charge, PI also interacts with the N-terminal region, contributing to α-Syn’s membrane-binding properties. It is the case of the negatively charged phosphatidylinositol 4,5-bisphosphate (PIP₂), which interacts with α-Syn’s N-terminal residues 1–10, facilitating helical conformation adoption and membrane localization through electrostatic contacts (Jacob et al., 2021). Additionally, cardiolipin, a mitochondrial-specific PL, has been implicated in modulating α-Syn’s oligomerization (Ghio et al., 2016). Studies suggest that cardiolipin not only enhances α-Syn’s binding affinity to mitochondrial membranes but also promotes the formation of pore-like structures by α-Syn oligomers (Ghio et al., 2019; Nakamura et al., 2011; Ryan et al., 2018).

Polyunsaturated fatty acids (PUFAs) are known to influence LDs size. Polyunsaturated fatty acids (PUFAs), such as docosahexaenoic acid (DHA) and α-linolenic acid (ALA), bind to the N-terminal domain of α-Syn and can influence its oligomerization by inducing conformational changes (Assayag et al., 2007; Broersen et al., 2006; De Franceschi et al., 2011, 2017; Fecchio et al., 2018; Galvagnion, 2017). α-Syn is reported to play a role in cholesterol metabolism. Its overexpression is associated with an increase of TGs and SEs (more specifically cholesterol ester) levels, related to an accumulation of LDs (Alza et al., 2021). Additionally, 27-hydroxycholesterol induces the expression and accumulation of α-Syn in dopaminergic neurons (Bell and Vendruscolo, 2021).

α-Syn membrane interactions are strongly influenced by lipid composition, membrane curvature, and the lipid-to-protein ratio, factors that are critical for both its normal physiological roles and its pathological behavior in diseases such as PD (Galvagnion et al., 2015; Kiechle et al., 2020; Pranke et al., 2011).

The phospholipid composition of LDs alone may not support strong α-Syn binding to the LD membrane; however, the presence of cholesterol appears to be a critical factor in promoting these interactions. Additional interactions with LD-associated proteins may also contribute. Recent studies indicate that α-Syn can bind cooperatively to phospholipid membranes, with a notably higher affinity for membranes that also contain proteins, a phenomenon that may likewise occur with LDs (Makasewicz et al., 2021).

Evidence for α-Syn interaction with LDs comes from studies showing its ability to bind both artificial and natural LDs. For instance, increasing the fatty acid concentration in cells, such as HeLa cells, has been shown to promote the translocation of α-Syn from the cytosol to the LD membrane (Cole et al., 2002; Thiam et al., 2013a). As a phospholipid sensor, α-Syn is naturally drawn to phospholipid membranes. Owing to its high lipid affinity, it also shows a pronounced tendency to interact with LDs. This preference is partly due to the LD monolayer being less densely packed with phospholipids, thereby exposing underlying neutral lipids. The hydrophobic nature of these lipids further facilitates α-Syn binding (Caillon et al., 2020; Kory et al., 2016; Thiam et al., 2013a; Thiam et al., 2013b).

### 3.2 LDs in the context of PD

**Figure 6:**
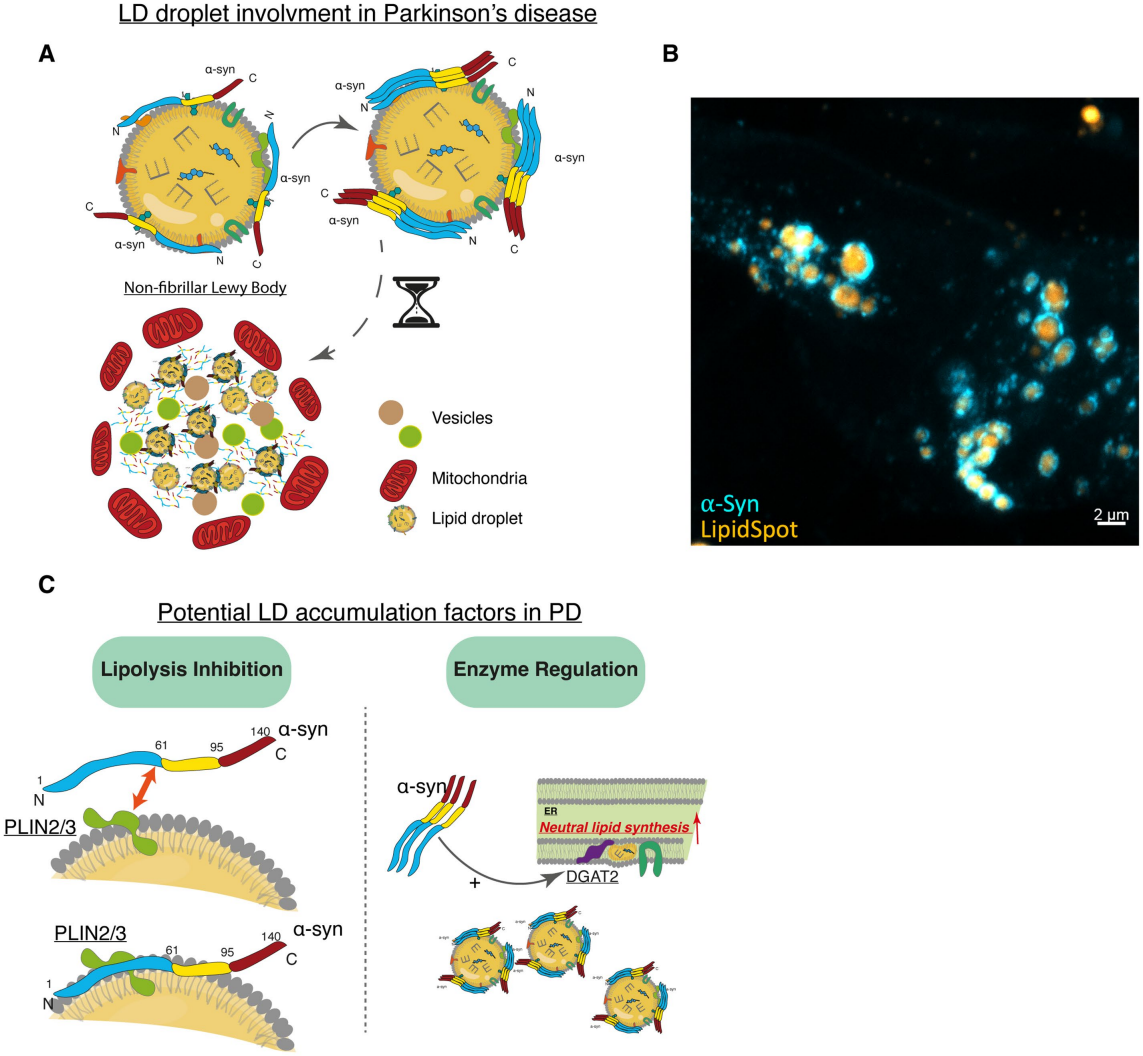
LDs involvement in PD. **(A)** Illustration of the interaction of LDs and α-Syn leading to its aggregation and formation of non-fibrillar LBs, represents a hypothetical model. **(B)** Confocal image of α-Syn aggregates engulfing LDs. α-Syn (Cyan Blue), LDs (gold-yellow). Scale bar = 2 μm. **(C)** Potential LD accumulation factors: Lipolysis Inhibition: α-Syn cooperates with LD-coating proteins like Perilipin 2 and 3 (PLIN2 and PLIN3) to inhibit lipolysis, potentially altering lipid homeostasis in neurons. Enzyme Regulation: α-Syn overexpression upregulates DGAT2 and activates ACS, triggering LD synthesis.

Many studies reveal a major role of lipids in PD pathogenesis, more precisely in oxidative stress response, and lysosomal dysfunction, showing the potential role of LD in LBs formation (Battis et al., 2023; Fanning et al., 2020; Galvagnion, 2017; Shahmoradian et al., 2019) (Figure 6A). In 2017, a genomic study revealed the major role of LDs in PD pathogenesis, as lipids and lipoproteins are involved in processes responsible for dopaminergic neurons’ death (oxidative stress, endo-lysosomal function, ER stress response and immune response activation) (Klemann et al., 2017). Different lipids have been found in synuclein-containing Lewy bodies purified from human PD brains (Yang et al., 2022).

LBs are not purely proteinaceous aggregates but consist of crowded membranous organelles, including vesicles, mitochondria, lysosomes, and lipid-rich structures (Fanning et al., 2020; Gai et al., 2000; Mahul-Mellier et al., 2020; Shahmoradian et al., 2019). Using correlative light and electron microscopy (CLEM), the study identifies α-Syn-immunopositive inclusions containing a mix of membranes, organelles, and lipids, suggesting impaired organellar trafficking as a potential driver of PD pathogenesis (Shahmoradian et al., 2019).

Moreover, Brekk et al. (2020), investigated the presence of LDs and, more specifically, neutral lipids in PD patients. This study revealed an accumulation of neutral lipids in dopaminergic neurons, localizing to α-Syn-rich inclusions. However, neutral lipid-enriched structures were not positive for PLIN2 staining, suggesting that these structures are not necessarily LDs, or hypothetically LDs with a modified structure and protein composition. Notably, the accumulation of neutral lipids in dopaminergic neurons is linked to increased lipid levels in microglia but reduced levels in astrocytes, indicating a broader disruption of lipid homeostasis in the brains of PD patients (Brekk et al., 2020). These findings also raise an important question regarding how LDs should be defined in pathological contexts. While canonical LDs are typically characterized by the presence of specific associated proteins such as PLIN2 and PLIN3 (Conte et al., 2022), the PLIN2-negative lipid inclusions reported in PD (Brekk et al., 2020) could reflect recruitment of alternative brain-enriched PLIN isoforms (e.g., PLIN3 or PLIN5), impaired LD-associated protein recruitment, or accumulation of non-mature LDs. Accordingly, it may be more appropriate to refer to these structures as “lipid-rich organelles” or “LD-like structures” where PLIN-coating is unconfirmed, acknowledging their potential divergence from physiological LDs.

Recently, the vulnerability of human stem cell-derived dopaminergic neurons was observed after the induced accumulation of glucocerebrosides. The accumulation of this lipid was associated with the upregulation of PLIN2, suggesting a link between the accumulation of LDs and vulnerability of dopaminergic neurons in PD (Russo and Riessland, 2024).

The relevance of LD dysregulation in PD is supported by multiple lines of human evidence. Postmortem lipidomic analyses reveal increased oleic acid, diglycerides, and cholesterol esters in PD brains particularly within dopaminergic regions and α-Syn rich inclusions (Brekk et al., 2020; Fanning et al., 2019; Fanning and Selkoe, 2025; Shahmoradian et al., 2019). Genetic studies further support a mechanistic link between LD biology and PD pathogenesis: *GBA* mutations promote to glucosylceramide accumulation and upregulate PLIN2 in iPSC-derived dopaminergic neurons (Russo and Riessland, 2024), *LRRK2* mutations alter phospholipid metabolism relevant to LD monolayer composition (Galper et al., 2022), and *SMPD1* mutations impair lysosomal lipid degradation, resulting in secondary LD accumulation (Alecu and Bennett, 2019; Fais et al., 2021). Moreover, postmortem substantia nigra tissue displays cell-type-specific lipid alterations, with increased neutral lipids in dopaminergic neurons and decreased levels in astrocytes (Brekk et al., 2020). Finally, elevated cerebrospinal fluid lipid species correlate with motor symptom severity in PD patients (Galper et al., 2022). Together, these findings establish LD dysregulation as an active and clinically relevant contributor to PD pathogenesis.

Furthermore, the role of LDs in PD pathogenesis is supported by various *in vitro* or *in vivo* studies, which demonstrate the interplay between LDs and α-Syn.

Under physiological conditions, α-Syn binds to LDs monolayer where it accumulates, protects from lipolysis and promotes LDs accumulation (Farmer et al., 2020). As previously mentioned, the initial accumulation of LDs is meant to be protective by sequestering fatty acids that could induce cellular stress (Olzmann and Carvalho, 2019). However, the excess of LDs and high level of peroxidized lipids can contribute to the formation of insoluble α-Syn aggregates. Indeed, studies have shown that the stimulation of LDs formation with oleic acid (OA), exacerbates α-Syn aggregation. This has been shown in the study by Fanning et al. (2019), where they observed an increase of α-Syn inclusions in mouse neuroblastoma cells expressing a mutated form of α-Syn (αS3K) (Fanning et al., 2019). These results correlate with a more recent study by Eubanks et al. (2025), where OA-induced LD formation is associated with morphological modifications of α-Syn aggregates toward a “Swiss cheese” that could be more resistant to degradation (Eubanks et al., 2025). These findings reinforce the idea that LD-α-Syn interactions induce proteolytic-resistant α-Syn conformers, as demonstrated in a Drosophila model of PD (Girard et al., 2021).

Moreover, it is known that an excess of α-Syn can disrupt lipid homeostasis and result in lipid peroxidation and ferroptosis, leading to the degeneration of dopaminergic neurons (Mahoney-Sanchez et al., 2022). In a Drosophila model of PD, α-Syn was found to localize on the surface of lipid droplets, where it cooperates with LD proteins to inhibit lipolysis and lead to the accumulation of lipid droplets. This was associated with an increase in the resistance of α-Syn to proteolytic digestion, promoting its aggregation (Girard et al., 2021). Interestingly, a recent study showed that LDs serve as substrates for protein phase separation, a process that can lead to the formation of amyloid fibrils, including those of α-Syn (Kamatar et al., 2024; Mukherjee et al., 2023; Ray et al., 2020). This implies that lipid droplets may contribute to the pathological aggregation of proteins in neurodegenerative diseases such as PD, paving the way for further research into the mechanisms underlying α-Syn aggregation. Cole et al. (2002), demonstrated that oligomers of α-Syn preferentially associate with LDs and cell membranes (Cole et al., 2002). This is also demonstrated in Figure 6B, where α-Syn aggregates accumulate on LDs membranes (Figure 6B). By accumulating on PL surfaces, these oligomers slow the lipolysis of LDs (Cole et al., 2002; Outeiro and Lindquist, 2003) (Figure 6C).

α-Syn has been found to selectively bind to cholesteryl-ester-rich LDs in multiple human cell lines and stem cell-derived dopaminergic neurons. This binding promotes the formation of α-Syn multimers and is highly temperature sensitive. Excess α-Syn stimulates LD accumulation, restricts organelle size, and ensures intracellular LD organization, which strictly depends on functional membrane-binding. Furthermore, once attached to LDs α-Syn becomes more mature and acquires a stable state. This interaction involves residues 1–100 of α-Syn and it’s important for regulating LDs size and abundance, suggesting a role in cellular lipid metabolism (Jacob et al., 2024).

The interaction between α-Syn and LDs plays a significant role in cellular lipid and cholesterol metabolism. Studies have shown that α-Syn overexpression leads to an increase in triacylglycerol (TAG) and cholesteryl ester (CE) levels, accompanied by LD accumulation. This accumulation is associated with changes in lipid metabolism enzymes, including upregulation of DGAT2 and activation of ACS that could be linked to PD (Alza et al., 2021) (Figure 6C).

A lipidomic study by Fanning et al. (2019), revealed an increase in oleic acid and diglycerides in yeast expressing human α-Syn. This increase was shown to be toxic for cells; however, LDs appeared to protect cells against this accumulation by sequestering oleic acid and diglycerides. In the same study, elevated oleic acid levels enhanced α-Syn toxicity, whereas inhibiting stearoyl-CoA desaturase, the enzyme responsible for oleic acid production, provided a protective effect (Fanning et al., 2019). Alterations in lipid metabolism, such as the accumulation of triacylglycerol and cholesteryl esters, have been observed in cells overexpressing α-Syn. These changes were linked to the activation of specific enzymes and regulatory proteins, which can lead to lipid droplet accumulation and potentially contribute to neuronal death (Alza et al., 2021).

In 2018, a study investigating PLIN4-dependent LDs’ role in PD, demonstrated by using a mouse model of PD, an accumulation of LDs in dopaminergic neurons. This excessive accumulation appeared to disrupt mitophagy, ultimately leading to neurodegeneration. These findings highlight the role of LD dysregulation and suggest that LD-associated markers could serve as potential biomarkers (Han et al., 2018).

Conversely, certain lipids have been shown to exert neuroprotective effects by modulating lipid droplet dynamics and enhancing autophagy. Linoleic acid (LA) has been shown to stimulate the biogenesis of LDs. This process is crucial because LDs play a significant role in cellular energy storage and metabolism, which are vital for maintaining cellular health and function, especially in neurons. Moreover, LA enhances autophagic flux and lipophagy, which are processes that help in the degradation and recycling of cellular components, including lipid droplets. This enhancement leads to an antioxidant effect, reducing oxidative stress (Alarcon-Gil et al., 2022). This suggests that targeting lipid metabolism could be a therapeutic strategy for PD.

**Figure 7:**
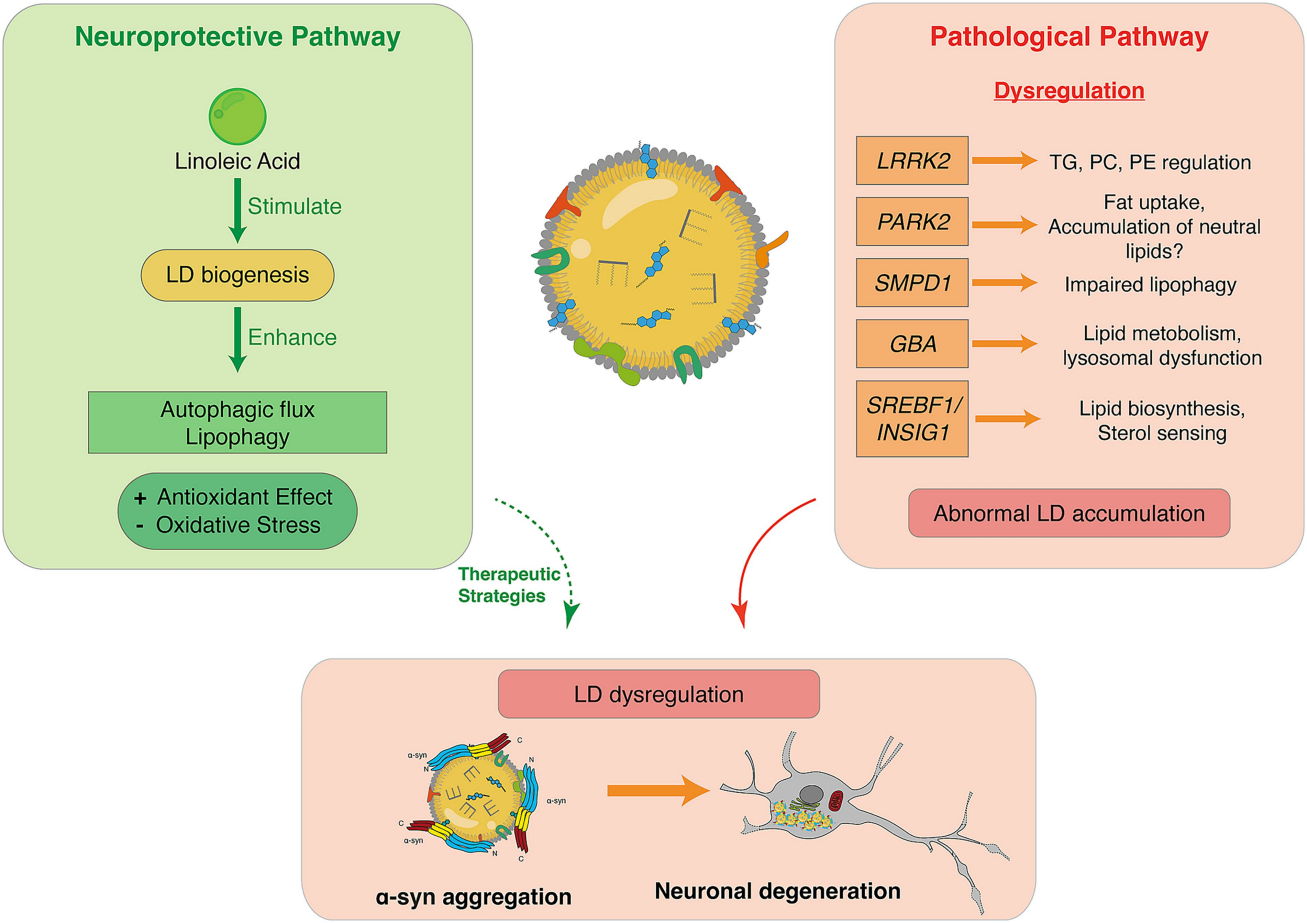
LDs dysregulation in PD. (Left panel) Schematic representation of the neuroprotective pathway, linoleic acid stimulates LD biogenesis and enhances autophagic flux and lipophagy, promoting antioxidant effects and reducing oxidative stress. (Right panel) Schematic representation of the pathological pathway involving gene dysregulation (*LRRK2, PARK2, SMPD1, GBA*, and *SREBF1/INSIG1*), resulting in abnormal LD accumulation, by impairing lipolysis, altering lipid metabolism, etc. (Bottom panel) Pathological pathway enhances α-Syn aggregation on the LD surface, leading to neuronal degeneration, linking LD abnormalities to the mechanisms of PD pathogenesis.

The mechanisms underlying the dysregulation of lipid homeostasis in PD are still not clear, but known genes or proteins like *LRRK2* or Parkin seem to play a crucial role in regulating neuronal lipids. Many genes and proteins directly or indirectly involved in lipid droplet biogenesis and regulation have been identified as risk factors in PD. Among them, *LRRK2* is one of the most associated with PD. Its regulatory role of lipids, including TGs, PC or even PE (Galper et al., 2022), could indirectly play on the composition of LDs. Parkin (*PARK2*), a key protein in PD, is known to regulate fat uptake, influencing cellular lipid storage and potentially favorising the accumulation of neutral lipids (Kim et al., 2011). Also, some pathways involving Parkin may be activated by oxidative stress, influencing LD formation and neuronal survival (Tang et al., 2023). Mutations in *SMPD1*, which encodes acid sphingomyelinase, result in lysosomal dysfunction and impaired lipid degradation, potentially leading to LD accumulation (Alecu and Bennett, 2019; Fais et al., 2021). The *GBA* gene, regulating glycosphingolipid metabolism, is also associated with lysosomal dysfunction and presents a PD risk as well as an indirect LD regulator (Alecu and Bennett, 2019). Finally, genes like *SREBF1*, encoding SREBP, a transcription factor responsible for lipid biosynthesis, and *INSIG1*, which regulates sterol sensing and SREBP activation, are also relevant to PD and directly involved in LD biogenesis (Do et al., 2011; Elhadi et al., 2025; Tang et al., 2023). These genes could contribute to α-Syn aggregation in PD, and their dysregulation offers mechanistic insights into PD pathology (Figure 7). The significance of LDs in functions as lipid storage and metabolism brings new insights into PD understanding.

One of α-Syn mutations, A53T, has been described in various studies as involved in α-Syn association to LDs, inducing an increase of TG production and LD accumulation in dopaminergic neurons (Vinueza-Gavilanes et al., 2020). Moreover, neurons overexpressing α-Syn once exposed to iron showed an increase in their TG and LD content (Sánchez Campos et al., 2018; Sere et al., 2010). Iron accumulation is indeed an event occurring in PD, interestingly associated with lipid peroxidation and LDs accumulation (Salvador, 2010; Sánchez Campos et al., 2015; Schneider and Bhatia, 2012). Finally, *GBA* variant E326K, which does not lead to a significant loss of GCase activity, represents a risk factor for PD and is also associated with an increase in insoluble α-Syn and accumulation of LDs (Smith et al., 2023).

The interplay between lipid droplets and α-Syn presents a promising area of research with significant implications for understanding the pathogenesis of PD and related disorders. Targeting lipid metabolism or enhancing lipid droplet function may offer novel therapeutic strategies to mitigate α-Syn toxicity and improve neuronal health (Bourdenx et al., 2021).

## 4 Discussion

This review highlights the emerging interplay between LDs and α-Syn in the pathogenesis of PD. While LDs have historically been perceived as inert lipid reservoirs, recent research has unveiled their different roles in cellular stress response, lipid metabolism, protein homeostasis and other functions (Olzmann and Carvalho, 2019; Walther and Farese, 2012). These functions are particularly critical in the central nervous system, where neuronal health is dependent on lipid metabolism and stress mitigation.

A growing body of evidence demonstrates that LDs could be active contributors to neurodegenerative processes (Farmer et al., 2020; Marschallinger et al., 2020). Notably, several studies revealed that LDs interact directly with α-Syn, a key pathological hallmark of PD (Cole et al., 2002; Eubanks et al., 2025; Jacob et al., 2024; Outeiro and Lindquist, 2003). This relationship seems to be bidirectional: α-Syn promotes LD accumulation by modulating lipid metabolism and inhibiting lipolysis (Girard et al., 2021), while LDs provide a surface that enhances α-Syn aggregation (Eubanks et al., 2025; Jacob et al., 2024). One key mechanism is that α-Syn preferentially binds to LD membranes enriched with specific lipids, such as cholesterol esters and phospholipids with packing defects (Jacob et al., 2024; Jakubec et al., 2021; Johnson et al., 2025). The propensity of α-Syn to interact with such LD membranes may drive the formation of α-Syn aggregates, forming proteolysis-resistant inclusions (Girard et al., 2021; Kamatar et al., 2024).

Importantly, lipidomic studies have revealed alterations in lipid composition during PD, including increased levels of oleic acid and diglycerides. This could be an important factor of toxicity when not properly sequestered by LDs (Fanning et al., 2019). These findings align with reports of disrupted lipid homeostasis and ER stress in PD models, potentially driven by α-Syn-mediated LD accumulation (Brekk et al., 2020; Shahmoradian et al., 2019). While LD accumulation may initially serve a protective role against lipotoxicity and oxidative stress (Ackerman et al., 2018; Bensaad et al., 2014), chronic or excessive accumulation contributes to mitochondrial dysfunction, impaired autophagy, and ferroptosis which are pathological features commonly observed in PD (Han et al., 2018; Mahoney-Sanchez et al., 2022).

A critical distinction is whether LD accumulation initiates pathology or reflects compensatory lipid sequestration. Evidence indicates that both mechanisms are stage-dependent. Early LD formation can be protective, sequestering peroxidized lipids and free fatty acids to mitigate oxidative stress (Ackerman et al., 2018; Bensaad et al., 2014). However, temporal studies show that α-Syn overexpression induces LD accumulation within hours to days, often preceding detectable mitochondrial or lysosomal dysfunction (Alza et al., 2021), suggesting that α-Syn can directly drive LD remodeling.

Once established, chronic LD accumulation shifts from a protective to pathogenic state, disrupting mitochondrial dynamics and impairing autophagy (Girard et al., 2021; Han et al., 2018). This shift initiates a vicious cycle in which LD buildup impairs mitochondrial fission and autophagy, increasing oxidative stress and further promoting LD biogenesis (Marschallinger et al., 2020).

To dissect these temporal relationships, models with fine control over α-synuclein aggregation, such as LIPA (Light-Inducible Protein Aggregation)-α-Syn, are needed to precisely determine when LD remodeling becomes maladaptive (Bérard et al., 2022).

This distinction carries therapeutic implications: if LD accumulation is initially compensatory, blocking LD formation could exacerbate lipotoxicity; conversely, if chronic accumulation drives neurodegeneration, enhancing lipophagy may be neuroprotective. Modulating the expression of associated proteins, such as perilipins or activating lipophagy, represents promising approaches to restore lipid homeostasis in PD. Supporting this, linoleic acid supplementation promotes LD biogenesis while simultaneously stimulating autophagy/lipophagy and reducing oxidative stress in PD models (Alarcon-Gil et al., 2022). Additionally, the temperature-sensitive interaction of α-Syn with cholesteryl-ester-rich LDs suggests that α-Syn actively senses and regulates LD composition, rather than being a passive bystander (Jacob et al., 2024).

Future work integrating high-temporal-resolution imaging, multi-omics profiling, combined with innovative α-Syn aggregation models like LIPA, will be critical to resolving causality and identifying optimal therapeutic intervention windows.

The complex interplay between LDs and α-Syn represents a novel and promising research area in PD pathology. Evidence suggests that α-Syn not only regulates LD formation and composition but is also sequestered by LDs, promoting its aggregation and toxicity under pathological conditions. Further elucidation of the LD-α-Syn relationship could open the way for new therapeutic modulating lipid metabolism, enhancing lipid droplet turnover, and mitigating neurodegeneration. Future research should focus on *in vivo* studies, high-resolution imaging of LD-α-Syn interactions, and the development of interventions targeting lipid-protein interactions to better understand PD.
